# The overload loop: a distinct reoxygenation pattern above the second ventilatory threshold revealed by a new analytical method

**DOI:** 10.5114/biolsport.2026.159557

**Published:** 2026-02-23

**Authors:** Annette Schmidt, Lucas Koch, Tom Brandt, Timo Schinköthe

**Affiliations:** 1 Sports Biology, University of the Bundeswehr Munich, Neubiberg, Germany; 2 Research Center Smart Digital Health, University of the Bundeswehr Munich, Neubiberg, Germany

**Keywords:** NIRS, SmO_2_ pacing, High-intensity exercise, Ventilatory threshold, Muscle oxygenation

## Abstract

Overpacing is frequently observed in high-intensity functional training modalities such as CrossFit, where athletes exceed sustainable intensity domains early and subsequently fail to recover performance within the same exercise bout. This study examined whether exercising above the second ventilatory threshold (VT2) induces distinct reoxygenation patterns in active and inactive muscles and aimed to develop a mathematical method for quantifying these intensity-dependent effects. Fifty-four healthy men performed two incremental cycling tests, one above and one below VT2, while SmO_2_ of the vastus lateralis and triceps brachii was continuously measured. Heart rate served as a systemic reference to align local SmO_2_ values, yielding the new metric $MUSCLE\frac{\Delta SmO_{2}}{\text{HRrel}}$, defined as the largest exercise-to-recovery difference at identical relative heart rates. Brachial artery diameter was additionally assessed in a subsample. Only above VT2 did the inactive muscle continue to deoxygenate into early recovery, whereas the active muscle reoxygenated immediately. When plotting both muscles against each other, this produced a distinct circular overload loop, not observed below VT2. $MUSCLE\frac{\Delta SmO_{2}}{\text{HRrel}}$ confirmed this asymmetry statistically, showing a large effect in the triceps brachii (p < 0.001) and no meaningful difference in the vastus lateralis. Above VT2, brachial artery diameter decreased and subsequently increased during recovery, supporting intensity-dependent vascular regulation. Exceeding VT2 triggers an asynchronous reoxygenation response between muscle groups. The overload loop and its quantification using the newly developed metric provide a novel tool for analyzing intensity-driven SmO_2_ dynamics and offer new insight into the coordination of local and systemic vascular regulation under conditions of unsustainable exercise intensity.

## INTRODUCTION

High-intensity functional training modalities such as CrossFit require athletes to self-regulate exercise intensity under conditions of high metabolic demand and rapidly changing movement patterns [1–3]. A frequently observed phenomenon in this context is overpacing, defined as an excessively high initial exercise intensity that cannot be sustained throughout the workout [4, 5]. Overpacing typically results in a rapid decline in performance and, critically, an inability to re-establish a stable work rate despite deliberate reductions in external load or movement speed [6, 7].

This persistent impairment suggests that overpacing reflects a qualitative alteration in physiological regulation rather than a gradual accumulation of fatigue alone, consistent with the loss of steadystate control observed at high exercise intensities [7, 8]. From a physiological standpoint, overpacing may be interpreted as a premature transition into intensity domains above the second ventilatory threshold (VT2), a range in which steady-state conditions are no longer attainable [7, 9]. Exceeding VT2 is associated with pronounced sympathetic activation, progressive metabolic disturbance, and intensity-dependent alterations in the regulation of regional blood flow and oxygen delivery [10]. While these mechanisms account for the initial performance decline, they do not fully explain why recovery fails to occur within the same exercise bout, particularly in multimodal exercise formats involving alternating active and inactive muscle groups.

A mechanistic understanding of overpacing, therefore, requires methodological approaches capable of capturing intensity-dependent alterations in muscle oxygenation and reoxygenation dynamics across different muscle groups, providing insight into the coordination between systemic physiological stress and local oxygen supply. Given that overpacing may reflect intensity-dependent disruptions in the coordination of systemic stress and regional oxygen supply, it is essential to distinguish between active and accessory muscles when analyzing muscle oxygenation dynamics during exercise and recovery [11, 12].

Incremental cycling provides a controlled experimental model to induce systemic metabolic stress while enabling simultaneous observation of muscle regions with high locomotor involvement and those that remain largely inactive. Within this framework, the vastus lateralis (VL) is a primary locomotor muscle, with activation closely linked to power output and oxygen consumption. In contrast, upper-body muscles primarily function as accessory tissue under laboratory conditions [13–15]. In upper-body muscles that functioned solely as accessories, only low muscle activation was observed [16]. It has also been shown that oxygen uptake in the accessory muscles during cycling is minimal, contributing little to overall deoxygenation [17]. In line with previous literature, the terms ‘active’ muscle and ‘inactive’ muscle refer to those primarily responsible for locomotor function and those that do not contribute substantially [18, 19].

A commonly used parameter for evaluation is the half-recovery time (HRT). HRT describes the time a muscle requires to reach half of its individual reoxygenation amplitude after cessation of exercise and thus enables a robust assessment of the restoration of the balance between oxygen supply and demand. The methodological approach is based on identifying the minimal SmO_2_ (muscle oxygen saturation, expressed as %) at the end of the work phase and subsequently measuring the time required to reach half of the recovery amplitude, thereby enabling reliable comparisons across muscle groups [12]. Similar metrics, such as the ‘net time to half-recovery’ or ‘gross time to half-recovery’, are calculated either based on the time it takes from the minimum SmO_2_ value during or after exercise and 50% of the peak SmO_2_ value after exercise, or the time it takes between the end of exercise and 50% of the peak SmO_2_ value after exercise [11]. Active and inactive muscles consistently exhibit distinct reoxygenation patterns. Active musculature reoxygenates rapidly and immediately after exercise termination, resulting in short recovery values. Inactive musculature, by contrast, shows a delayed recovery and may even continue to deoxygenate before the reoxygenation process begins. The relationship between exercise load and recovery time has so far been described as uniform across increasing workloads by these mathematical methods, with no breakpoints observed [11, 12].

In this context, exercise intensity is not characterized by a continuous physiological response but by distinct breakpoints that reflect underlying metabolic transitions. These breakpoints manifest as systematic changes in the slope and curvature of muscle oxygen saturation (SmO_2_) trajectories as exercise intensity approaches and surpasses established physiological thresholds. Below the first ventilatory threshold (VT1), SmO_2_ typically stabilizes or may even increase slightly, indicating a balance between oxygen delivery and utilization. Surpassing VT1 marks the onset of progressive deoxygenation. In contrast, exercise intensities beyond the second ventilatory threshold (VT2) are characterized by a markedly steeper decline in SmO_2_, reflecting a sustained shift toward non-steady-state conditions and increasing reliance on anaerobic energy provision [20, 21]. Consistent with this interpretation, minimal SmO_2_ values attained during incremental exercise have been shown to correlate strongly with peak oxygen uptake, underscoring the close coupling between SmO_2_ dynamics and global physiological stress [22–24]. Importantly, these threshold-related transitions are intrinsic to the exercise phase itself and represent qualitative changes in physiological regulation. This contrasts with commonly used recovery-based metrics such as halfrecovery time (HRT), net time to half-recovery, or gross time to halfrecovery, which capture only post-exercise reoxygenation behavior and are inherently insensitive to threshold-dependent alterations occurring during exercise.

Accordingly, this work aimed to establish an experimental design that allows for observing the influence of VT2 on post-exercise reoxygenation in a controlled and reproducible manner. In parallel, the study aimed to develop a mathematical method to statistically assess the effect of exceeding VT2 on skeletal muscle reoxygenation behavior. The intended method should also enable a direct comparison between active and inactive muscles under identical relative physiological stress. By relating local SmO_2_ measurements to a systemic reference such as heart rate (HR), the planned approach was expected to offer a coherent framework for analyzing post-exercise reoxygenation across different muscle types.

## MATERIALS AND METHODS

### Experimental design

**FIG. 1:**
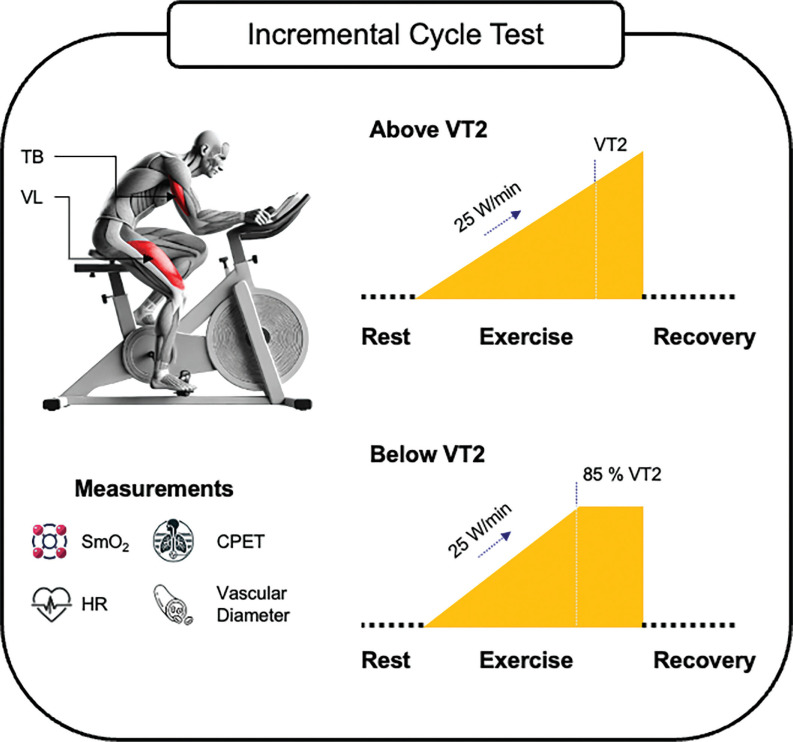
Schematic overview of the study. CPET, cardiopulmonary exercise testing; HR, heart rate; min, minute; SmO_2_, muscle oxygen saturation; TB, triceps brachii; VL, vastus lateralis; VT2, second ventilatory threshold; W, watts.

This study was conducted at the University of the Bundeswehr Munich (UniBw M). During two consecutive incremental cycling tests, the SmO_2_ of an active muscle (VL) and an inactive muscle (TB) were measured continuously in combination with cardiopulmonary exercise testing (CPET). The tests were performed under laboratory conditions and in the same order, first as ‘test above VT2’ followed by ‘test below VT2’ with a rest period of at least 48 hours (h) between test sessions. Processing and use of data has been carried out in accordance with the General Data Protection Regulation (GDPR, 2016/679). The trial was registered on ClinicalTrials.gov (NCT06884644). An overview of the study design is displayed in Figure 1.

### Subjects

The inclusion criteria were defined as follows: subjects had to be (a) 18 years of age or older, (b) healthy with no chronic or acute physical or mental illnesses (especially cardiovascular, pulmonary, muscular system) and no use of prescription drugs affecting the metabolism or the cardiovascular system (e.g. beta-blockers or antidepressants), no exercise-induced asthma and (c) without tattoos in the areas of the sensor placements. 54 male subjects between 19 and 58 years of age participated in this study (Table 1). Subjects were recruited regardless of cycling background or training status. The sample included therefore both recreationally active and sedentary individuals.

**TABLE 1:** Subject characteristics

Variable	Incremental cycling test
Subjects	n = 54 (all male)
Age (yrs)	25.0 ± 7.2
Height (m)	181.1 ± 6.6
Weight (kg)	83.4 ± 12.2
BMI (kg/m^2^)	25.4 ± 3
FM (%)	15.7 ± 5
FFM (%)	84.3 ± 5
RHR (bpm)	80.1 ± 14.9^†^
HRmax (bpm)	185.9 ± 10.4^†^
${\dot{\text{V}}\text{O}}_{2\max}$ (ml/kg/min)	49.0 ± 7.8^†^

† In reference to the test above VT2; Values are presented as mean ± standard deviation (SD); BMI, body mass index; bpm, beats per minute; FFM, fat-free mass; FM, fat mass; HRmax, maximum heart rate; kg, kilogram; m, meter; min, minute; ml, milliliter; RHR, resting heart rate; ${\dot{\text{V}}\text{O}}_{2\max}$, maximum oxygen uptake; VT2, second ventilatory threshold; yrs, years.

The Institutional Ethics Committee of the UniBw M approved the study protocol, ensuring that it conformed to the ethical guidelines of the 2024 Declaration of Helsinki (30/05/2023, EK UniBw M 23–40). Subjects were informed about the purpose, procedures, and risks in this study and provided written informed consent.

### Procedure

Demographic and anthropometric data were collected using a questionnaire and bioelectrical impedance analysis (BIA) prior to the incremental cycling test. Subjects were instructed to refrain from any intensive physical activity 48 h, alcohol consumption 24 h, and food intake 3 h before the test session and do not drink more than 500 milliliters of water 30 min before the measurement.

For the incremental cycling test above VT2, subjects performed a ramped incremental load test on a bicycle ergometer. The test began with a 1-minute rest followed by a 3-minute warm-up at 50 watts (W). Subjects had to maintain a cadence of at least 60 revolutions per minute (rpm). After the warm-up, load was increased by 25 W per minute until exhaustion defined as the point at which a cadence of 60 rpm could no longer be maintained or when the subjects themselves indicated exhaustion. Subsequently, subjects rested for 7 minutes while sitting on the bicycle ergometer without pedaling. A particularly motivated and compliant subject was selected from the sample to undergo additional measurements of the vascular diameter of the brachial artery using sonography. The subject completed the identical incremental cycling test a total of six times on separate days.

During the second test session, subjects performed the incremental cycling test below VT2. The same test protocol was applied, except that the load increase was stopped at the wattage corresponding to 85% of the HR at VT2 from the previous test above VT2. 85% was selected to keep the load close to VT2 while accounting for natural HR fluctuations, which make precise maintenance challenging. Then, subjects continued cycling at a steady-state load for the same duration as in the test above VT2, followed by a 7-minute recovery period.

The protocol we used for testing above VT2 was based on established parameters, whereby an initial load of 50 W with a warm-up period of 3 minutes and an increase of 25 W/min during the exercise proved to be suitable for maximum testing across subjects of varying fitness levels, but also for submaximal loads [25, 26]. A ramp protocol was chosen because it shows gradual changes in ventilation, HR, and SmO_2_ dynamics—key variables in our study—as well as enabling more accurate VT2 detection compared to the step protocol [27].

The protocol below VT2 was completely purpose-built and tailored to our research question, whereby we were particularly concerned with a) selecting an intensity level just below VT2 and b) measuring the precise exercise duration and recovery time as in the test above VT2.

### Equipment

#### Bioelectrical impedance analysis

Body composition was measured with a bioelectrical impedance analysis (BIA; mBCA 515 scale, seca GmbH & Co., KG, Hamburg, Germany). The BIA was validated for the measurement of body composition and has a high measurement accuracy [28].

#### CPET

A breath-by-breath analysis of the respiratory gases was carried out using a CPET (Quark CPET, COSMED S.r.I., Rome, Italy). Quark CPET is considered a valid measurement method for respiratory gas analyses [29], with a mean percent error of 3.5% for VO_2_ and 2.0% for ventilation (VE) [30]. In addition, the recording software OMNIA enabled the connection to an external HR monitor and controlled the resistance on the bicycle ergometer (MONARK LC6, Monark Exercise AB, Vansbro, Sweden).

#### Heart rate monitor

HR was assessed continuously with an HR monitor (smartLAB hrm W, HMM Diagnostics GmbH, Heddesheim, Germany), which was, connected to the OMNIA software.

#### NIRS sensors

SmO_2_ was measured continuously in the TB and VL using NIRS sensors (Moxy muscle monitor, Fortiori Design LLC, Minnesota, USA). During incremental cycling, Moxy Monitor sensors showed high reliability at low to moderate intensities (ICC = 0.773–0.992; SROC = 0.834–0.980), with increasing variability at higher intensity levels [31]. The sensors captured data at a sampling frequency of 0.5 Hz by averaging approximately 80 wavelength cycles, producing one data point every two seconds. The sensor’s firmware automatically applied a 5-second moving average smoothing filter. The sensor was calibrated internally by the device [32, 33].

The sensor placement on the VL and TB was guided by palpation following anatomical landmarks [34]. The sensor for the VL was placed at 2/3 on the line from the anterior spina iliaca superior to the lateral side of the patella. The sensor for the TB was placed at 50% on the line between the posterior crista of the acromion and the olecranon at 2 finger widths lateral to the line [35].

#### Ultrasound device

The brachial artery as the conduit artery of the TB was examined using an ultrasound device (Versana Premier, GE Healthcare GmbH KG, Solingen, Germany). We used the L6-12 transducer, which operates in a frequency range of 4–13 MHz which is suitable for superficial and peripheral vascular applications due to its high spatial resolution [36]. A measurement bias of only 0.011 mm, with a resolution limit of ~0.05 mm caused by pixel size was reported for diameter measurements with comparable devices [37], even though these values are obtained using automated edge detection software. We used a manual caliper-based method, however, with a standardized measurement protocol repeated at each time point and averaged values, which allows us to assume sufficient internal consistency and a reliable approximation of the diameter of the brachial artery.

The transducer prepared with ultrasound gel was placed at the inferior border of the axilla of the right arm, which was abducted by approx. 45 degrees, with a slight flexion in the elbow joint, with the subject sitting on the bicycle ergometer in an upright position. At each measurement point, 3-second sonographic video recording were carried out. The vascular diameter was calculated manually using the image-analysis tools of the operating software. Since three measurements were carried out in each of the two exercise phases, the respective mean was calculated for each phase. To ensure that the transducer was attached quickly and precisely at each measurement point without deviating from the other measurement positions, the subject was equipped with a shoulder orthosis (DONJOY Ultrasling Quadrant, Ormed GmbH, Freiburg, Germany).

#### Data Extraction

The NIRS sensors measured on average every 2 seconds, with occasional shorter or longer intervals. Although the sensors were started at the same time, there were software-related fluctuations in the raw data. Respiratory variables and HR were recorded during CPET at a sampling rate of 2 seconds, with a few variations. Due to the different sampling rates between NIRS sensors, HR monitor, and CPET and separate software, raw data were stored in various formats and time intervals. All raw data were processed and merged into a standardized one-second time grid. Missing values were interpolated using the last measured value. The combined data frames were displayed and analyzed in R version 4.4.3, with RStudio version 2024.12.1+563.

The CPET data were determined by a trained, experienced researcher with extensive experience in deciding ventilatory thresholds [38]. The following procedure was carried out based on the classic nine-panel plot [39] and was assessed using OMNIA software.

VT1 was determined at the point where:

a) Carbon dioxide production (VCO_2_) vs. oxygen uptake (VO_2_) increased in slope greater than 1 (V-slope method) [40, 41]
b) Ventilatory equivalents of oxygen (VE/VO_2_) increased continuously, not accompanied by an increase in the ventilatory equivalents of carbon dioxide (VE/VCO_2_) [40, 42, 43]
c) An increase in end-tidal O_2_ (PETO_2_) without a corresponding decrease in end-tidal CO_2_ (PETCO_2_) [40, 42, 43]

VT2 was determined at the point where:

a) Minute ventilation (VE) disproportionately increased vs. VCO_2_ [9, 43]
b) VE/VCO_2_ increased [9, 43, 44]
c) PETCO_2_ began to decrease [9, 43, 44]

#### Statistical Analyses

A new metric was developed to determine SmO_2_ dynamics in muscles during recovery. HR was incorporated into the calculation to include a systemic parameter alongside SmO_2_ values as local parameters. To enable interindividual comparability between subjects regardless of cardiovascular fitness levels and to avoid dependence on fixed time frames, the respective HR at a specific time point in the exercise test (HR_current_) was set in relation to the subject’s individual HR at VT2 (HR_VT2_), resulting in a relative HR (HRrel) that reflects a particular stress level:

$$HRrel=\frac{HR_{current}}{HR_{VT2}}$$

Based on this, the difference was calculated for each pair of SmO_2_ values that had the same HRrel during both exercise and recovery:

MUSCLEHRrelΔSmO2=MUSCLEHRrelRec−MUSCLEHRrelExe

**FIG. 2:**
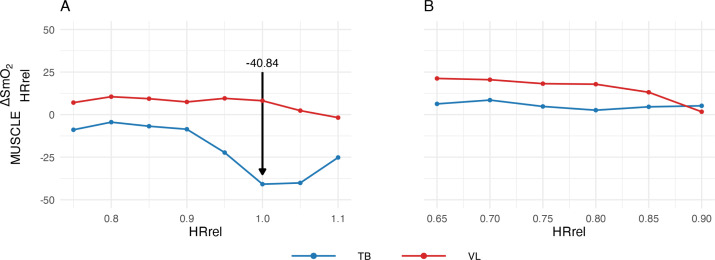
Representative subject courses of $MUSCLE\frac{\Delta SmO_{2}}{\text{HRrel}}$ in TB and VL of the incremental cycle test on a bicycle. (A) at the test above VT2; (B) at the test below VT2; HRrel, ratio of the current heart rate to the heart rate at the VT2; TB, triceps brachii; VL, vastus lateralis; VT2, second ventilatory threshold

The lowest value of $MUSCLE\frac{\Delta SmO_{2}}{\text{HRrel}}$ (i.e. the most negative value) found within a subject resulted in the variable $MUSCLE\frac{\Delta SmO_{2}}{\text{HRrel}}$. $MUSCLE\frac{\Delta SmO_{2}}{\text{HRrel}}$ represents the largest absolute difference (despite the negative sign) in SmO_2_ values between recovery and exercise for the respective muscle, and reflects the extent of SmO_2_ dynamics during recovery. $MUSCLE\frac{\Delta SmO_{2}}{\text{HRrel}}$ was determined for both investigated muscles and all conditions (above VT2; below VT2). The abbreviation of the muscle examined replaces ‘MUSCLE’. The course of $MUSCLE\frac{\Delta SmO_{2}}{\text{HRrel}}$ is shown in Figure 2. To improve comprehensibility, an example calculation for the representative subject in Figure 2 is presented in Table 2.

**TABLE 2:** Exemplary step-by-step calculation process based on the individual SmO_2_ values of the triceps brachii and vastus lateralis in exercise and recovery at predefined relative heart rate values to the maximum difference in SmO_2_

HR_current_ (bpm)	HRrel	$TB\frac{\text{Exe}}{\text{HRel}}$ (%)	$TB\frac{\text{Rec}}{\text{HRel}}$ (%)	$TB\frac{\Delta SmO_{\text{2}}}{\text{Max}}$ (%)	$VL\frac{Exe}{\text{HRel}}$ (%)	$VL\frac{\text{Rec}}{\text{HRel}}$ (%)	$VL\frac{\Delta SmO_{\text{2}}}{\text{Max}}$ (%)
118	0.75	63.80	54.89	-8.91	61.78	68.84	7.06

126	0.80	63.49	59.06	-4.43	60.78	71.31	10.53

133	0.85	62.74	55.92	-6.82	60.13	69.49	9.36

141	0.90	59.37	50.79	-8.58	57.86	65.33	7.47

149	0.95	52.74	30.46	-22.28	55.28	64.85	9.57

157 (=HR_VT2_)	1.00	49.30	8.46	**-40.84** (= $TB\frac{\Delta SmO_{\text{2}}}{\text{Max}}$)	48.63	56.79	8.16

165	1.05	42.60	2.49	-40.11	40.49	42.85	2.36

173	1.10	33.04	7.86	-25.18	36.79	35.03	**-1.76** (= $VL\frac{\Delta SmO_{\text{2}}}{\text{Max}}$)

bpm, beats per minute; HR, heart rate; HR_current_, current HR of the subject at a certain timepoint in the incremental cycling test; HRrel, ratio of the HR_current_ to the HR_VT2_; HR_VT2_, HR at the VT2; SmO_2_, muscle oxygen saturation; TB, triceps brachii; $TB\frac{\Delta SmO_{\text{2}}}{\text{Max}}$, difference of the SmO_2_ of the TB at identical HR values in ratio to the VT2; $TB\frac{\text{Exe}}{\text{HRel}}$, SmO_2_ of the TB at a specific HR ratio to the HR of the VT2 in the exercise; $TB\frac{\text{Rec}}{\text{HRel}}$, SmO_2_ of the TB at a specific HR ratio to the HR of the VT2 in the recovery; $TB\frac{\Delta SmO_{\text{2}}}{\text{Max}}$, maximal difference of the SmO_2_ of the TB at identical HR values in ratio to the VT2; $TB\frac{\Delta SmO_{\text{2}}}{\text{Max}}$, difference of the SmO_2_ of the VL at identical HR values in ratio to the VT2; $VL\frac{\text{Exe}}{\text{HRel}}$, SmO_2_ of the VL at a specific HR ratio to the HR of the VT2 in the exercise; $VL\frac{\text{Rec}}{\text{HRel}}$, SmO_2_ of the VL at a specific HR ratio to the HR of the VT2 in the recovery; $TB\frac{\Delta SmO_{\text{2}}}{\text{Max}}$, maximal difference of the SmO_2_ of the VL at identical HR values in ratio to the VT2; VL, vastus lateralis; VT2, second ventilatory threshold.

The data were tested graphically (histogram, Q-Q plot) and using the Shapiro-Wilk test for normal distribution. A one-sided paired ttest was performed to analyze differences $MUSCLE\frac{\Delta SmO_{2}}{\text{HRrel}}$ between the test conditions above and below VT2, as well as differences in vascular diameter of the brachial artery between phases. A one-sided t-test was chosen for the comparison of $MUSCLE\frac{\Delta SmO_{2}}{\text{HRrel}}$ between conditions, based on directional expectations derived from pilot data using a related protocol. Cohen’s d for paired samples was used to calculate the effect sizes [45]. 95% confidence intervals (CI) were provided for the descriptive means to express the uncertainty of the estimates. In accordance with the one-sided t-test a 90% CI was reported for the between-condition differences.

To determine the statistical sensitivity of the comparisons between conditions (above VT2 vs. below VT2) for the variables $TB\frac{\Delta SmO_{\text{2}}}{\text{Max}}$ and $VL\frac{\Delta SmO_{\text{2}}}{\text{Max}}$, a post hoc power analysis was performed using R (version 4.4.3). The statistical power was based on the effect sizes (Cohen’s d) of the respective paired-sample comparisons, for which a onesided t-test with a significance level of a = 0.05 was used. The estimated statistical power of the respective statistical test was thus derived from the observed effect sizes and sample sizes.

### RESULTS

**FIG. 3:**
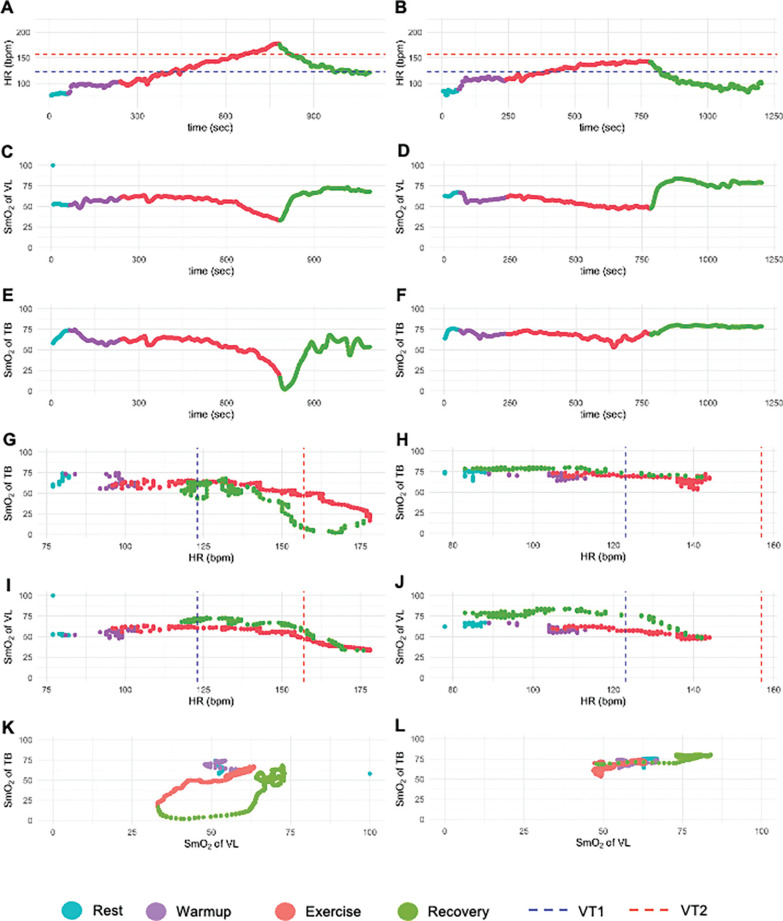
Representative subject courses of the incremental cycle test (A) at the test above VT2 with HR in relation to time; (B) at the test below VT2 with HR in relation to time; (C) at the test above VT2 with the SmO_2_ of the VL in relation to time; (D) at the test below VT2 with the SmO_2_ of the VL in relation to time; (E) at the test above VT2 with the SmO_2_ of the TB in relation to time; (F) at the test below VT2 with the SmO_2_ of the TB in relation to time; (G) at the test above VT2 with the SmO_2_ of the TB in relation to HR; (H) at the test below VT2 with the SmO_2_ of the TB in relation to HR (I) at the test above VT2 with the SmO_2_ of the VL in relation to HR (J) at the test below VT2 with the SmO_2_ of the VL in relation to HR. Bpm, beats per minute; HR, heart rate; Sec, seconds; SmO_2_, muscle oxygen saturation; TB, triceps brachii; VL, vastus lateralis; VT1, first ventilatory threshold; VT2, second ventilatory threshold.

The SmO_2_ dynamics of a representative subject – previously shown in the calculation example (Table 2) and the course of $MUSCLE\frac{\Delta SmO_{2}}{\text{HRrel}}$ (Figure 2) – are shown in Figure 3. The diagrams on the left side display the courses of variables in the test above VT2, on the right side similarly for the test below VT2. HR increases continuously both above VT2 (Figure 3A) and below VT2 (Figure 3B). Above VT2, it surpasses the HR at VT2, peaking at exhaustion before dropping in recovery. Below VT2, it plateaus at a lower HR than VT2 due to intensity stabilization and decreases immediately upon recovery. The SmO_2_ of the VL in the test above VT2 (Figure 3C) decreased during exercise, reaching its lowest point at exhaustion, followed by a rapid beginning of reoxygenation at recovery. In contrast, in the test below VT2 (Figure 3D), the VL first deoxygenated, then reached a steady state, and immediately reoxygenated at recovery.

**TABLE 3:** Numerical analysis for the occurrence of the overload loop by comparison of means of $TB\frac{\Delta SmO_{\text{2}}}{\text{Max}}$ and $VL\frac{\Delta SmO_{\text{2}}}{\text{Max}}$ between the test above and below VT2

Muscle	above VT2	below VT2	Difference between tests	Effect size (*d*)	p-value
$TB\frac{\Delta SmO_{\text{2}}}{\text{Max}}$ (%)	-23.60 [-27.32; -19.88]	-1.19 [-2.61; 0.23]	-22.41 [< -20]	-2.09	< 0.001
$VL\frac{\Delta SmO_{\text{2}}}{\text{Max}}$ (%)	2.34 [0.71; 3.98]	0.22 [-1.29; 1.72]	2.12 [< 3.45]	0.37	0.979

Values are presented as means [CI for “above VT2” and “below VT2” as 95%; CI for “Differences between tests” as 90% (from onesided paired t-test)]; HR, heart rate; SmO_2_, muscle oxygen saturation; TB, triceps brachii; $TB\frac{\Delta SmO_{\text{2}}}{\text{Max}}$, maximal difference of the SmO_2_ of the TB at identical HR values in ratio to the VT2; VL, vastus lateralis; $VL\frac{\Delta SmO_{\text{2}}}{\text{Max}}$, maximal difference of the SmO_2_ of the VL at identical HR values in ratio to the VT2; VT2, second ventilatory threshold.

The dynamics of the SmO_2_ of the TB showed a different course. In the test above VT2 (Figure 3E), deoxygenation occurred initially, but the lowest value was not reached at exhaustion. Recovery involved prolonged deoxygenation and delayed reoxygenation, a pattern only seen above VT2. In contrast, in the test below VT2 (Figure 3F), the TB SmO_2_ reached a steady state with immediate reoxygenation at recovery onset. Figures 3G, 3H, 3I, and 3J show SmO_2_ dynamics of both muscles plotted against HR, allowing comparison of physiological responses between exercise and recovery. Matched pairs of SmO_2_ values were displayed for each HR value, linking exercise and recovery. Except for the TB above VT2 (Figure 3G), reoxygenation during recovery was similar to or higher than during exercise.

Plotting the SmO_2_ dynamics of both muscles against each other revealed a circular pattern in the test above VT2 (Figure 3K), which is referred to as the ‘overload loop’. This visualization method enables graphical observation of the overload loop.

The overload loop is characterized by a prolonged decrease in SmO_2_ of the TB with the onset of the recovery, while the SmO_2_ of the VL immediately increased again. This SmO_2_ dynamics was observed only in TB and only after exercising at an intensity above VT2. Reoxygenation of the TB did not begin until the VL was either partially or almost completely reoxygenated.

The newly developed metric $MUSCLE\frac{\Delta SmO_{2}}{\text{HRrel}}$ enables this visual observation to be quantified, as it reflects the extent of the SmO_2_ dynamics of a muscle during recovery. The following descriptive mean values were obtained across all subjects, accompanied by a 95% confidence interval: $VL\frac{\Delta SmO_{\text{2}}}{\text{Max}}$ showed a 2.34% [0.71; 3.98] difference above VT2, which was not significantly different (p = 0.979, d = 0.37) from the 0.22% [-1.29; 1.72] difference below VT2. A post hoc power analysis confirmed sufficient sensitivity (1–b = 0.85, n = 54, a = 0.05 one-sided). In contrast, $TB\frac{\Delta SmO_{\text{2}}}{\text{Max}}$ showed a -23.60% [-27.32; -19.88] difference above VT2, significantly different (p < 0.001; d = -2.09) from the -1.19% [-2.61; 0.23] difference below VT2 (Table 3). A post hoc power analysis confirmed a sufficient sensitivity (1–b = 1.00; n = 54; a = 0.05; one-sided). $TB\frac{\Delta SmO_{\text{2}}}{\text{Max}}$ and $VL\frac{\Delta SmO_{\text{2}}}{\text{Max}}$ in the test below VT2 did not differ significantly (p = 0.156; d = -0.26) (Table 4).

**TABLE 4:** Differences between $TB\frac{\Delta SmO_{\text{2}}}{\text{Max}}$ and $VL\frac{\Delta SmO_{\text{2}}}{\text{Max}}$ in the test session below VT2

$TB\frac{\Delta SmO_{\text{2}}}{\text{Max}}$ below VT2	$VL\frac{\Delta SmO_{\text{2}}}{\text{Max}}$ below VT2	Difference between variables	Effect size (d)	p-value
-1.19 [-2.61; 0.23]	0.22 [-1.29; 1.72]	-1.41 [-3.37; 0.55]	-0.26	0.156

Values are presented as means [95% Confidence Interval]; HR, heart rate; SmO_2_, muscle oxygen saturation; TB, triceps brachii; $TB\frac{\Delta SmO_{\text{2}}}{\text{Max}}$, maximal difference of the SmO_2_ of the TB at identical HR values in ratio to the VT2; $VL\frac{\Delta SmO_{\text{2}}}{\text{Max}}$, maximal difference of the SmO_2_ of the VL at identical HR values in ratio to the VT2; VL, vastus lateralis; VT2, second ventilatory threshold

**FIG. 4:**
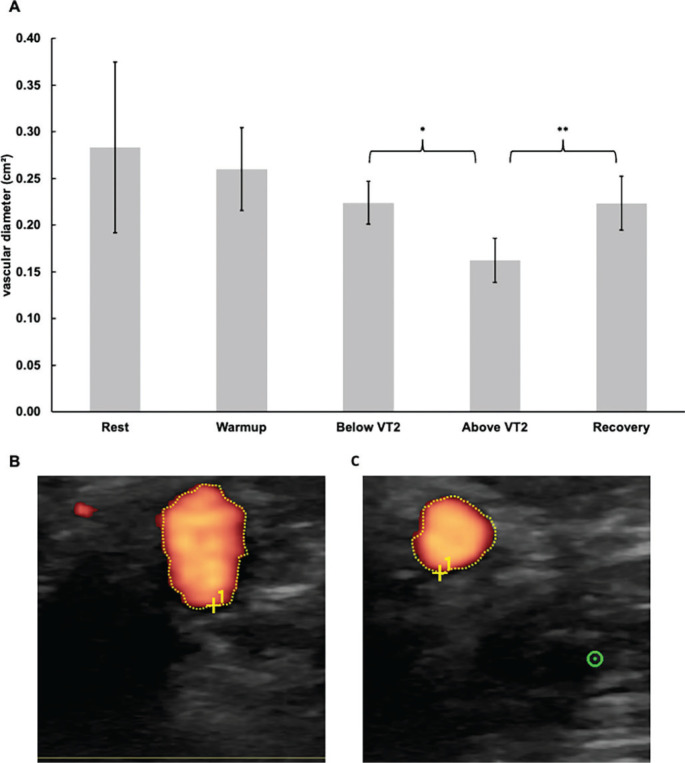
Sonography measurements above and below the VT2 and in the recovery. (A) course of the vascular diameter of the brachial artery (B) exemplary sonogram with the optical determination of the vascular diameter of the brachial artery below the VT2 (C) exemplary sonogram with the optical determination of the vascular diameter of the brachial artery above the VT2; * Indicates statistically significant differences at a level of p<0.05; ** Indicates statistically significant differences at a level of p<0.01; Error bars represent the 95 % confidence intervals; HR, heart rate; VT2, second ventilatory threshold.

The vascular diameter remained unchanged between rest, during warm-up, and below VT2. Exceeding VT2 caused a significant decrease (p = 0.013; d = -1.28) of 0.06 cm^2^. During recovery, the diameter significantly increased (p < 0.001; d = 2.77) by 0.06 cm^2^. The vascular diameter at each time point is shown in Figure 4.

## DISCUSSION

The present study investigated whether exceeding the second ventilatory threshold (VT2) induces characteristic SmO_2_ dynamics in active and inactive muscles and whether these responses can be quantified through a new mathematical approach. These findings are particularly relevant in the context of overpacing, a phenomenon commonly observed in high-intensity functional exercise, where athletes exceed sustainable intensity domains early and subsequently fail to recover performance within the same exercise bout [4–7]. Previous work demonstrated distinct differences in reoxygenation patterns between muscle groups: active muscles recovered rapidly, whereas inactive muscles showed prolonged deoxygenation and delayed reoxygenation after high-intensity exercise [11, 12]. However, the mathematical tools traditionally used to analyze these phenomena, such as half recovery time, net time to half recovery, and gross time to half recovery, describe recovery as a uniform process across increasing workloads and do not capture threshold-related inflection points during exercise. Consequently, they have not allowed systematic investigation of the influence of VT2 on post-exercise SmO_2_ behavior, nor have they allowed direct comparisons between active and inactive muscles under identical relative physiological stress.

In contrast to these previously established approaches [11, 12], the present study implemented an experimental design that specifically enabled the observation of SmO_2_ dynamics above and below VT2. The new method developed in this work aligned local SmO_2_ values with HR as a systemic reference to create an intensity equivalent comparison between exercise and recovery. This mathematical framework enabled quantification of the largest difference between SmO_2_ during exercise and during recovery at identical HRrel, yielding the new metric $MUSCLE\frac{\Delta SmO_{2}}{\text{HRrel}}$. By evaluating the matched SmO_2_ trajectories of the muscles across the same physiological stress levels, the method provides a statistically accessible representation of recovery behavior that is sensitive to changes associated with exceeding VT2.

Using this approach, a distinct pattern emerged in the inactive muscle above VT2, which was not observed below VT2 or in the active muscle. In the TB, deoxygenation continued into the early recovery phase, and reoxygenation was delayed until the VL had already reoxygenated almost entirely. When plotting the SmO_2_ values of both muscles against each other, this characteristic interplay formed a circular trajectory, which we describe as the ‘overload loop’. This visualization highlighted the asynchronous reoxygenation behavior of active and inactive muscles at intensities above VT2. Importantly, $MUSCLE\frac{\Delta SmO_{2}}{\text{HRrel}}$ provided an objective quantification of this phenomenon, confirming a large effect size above VT2 in the inactive muscle while showing no meaningful difference in the active muscle. These findings indicate that the overload loop is not only visually recognizable but also statistically substantiated through the newly introduced metric.

The physiological basis of this loop pattern likely reflects the coordinated but competing influences of sympathetic vasoconstriction and functional sympatholysis. Indeed, systemic sympathetic activity increases with exercise intensity, which can promote vasoconstriction in areas of low metabolic demand (e.g., inactive muscles). In contrast, in active muscle tissue, local metabolic signals attenuate this sympathetic vasoconstriction, a phenomenon known as functional sympatholysis [46]. Above VT2, such sympathetic activation is expected to be particularly pronounced, favoring vasoconstriction in inactive muscles. At the same time, high metabolic stress in the active muscle may trigger functional sympatholysis, thereby enabling local vasodilation and prioritizing oxygen delivery to the working tissue [46]. This imbalance could thus favor reoxygenation of the active muscle at the expense of the inactive muscle, which continues to experience reduced perfusion and, consequently, deoxygenation despite the cessation of exercise. The concept that blood flow redistribution during exercise is mediated by a combination of systemic sympathetic restraint and local metabolic vasodilation has been described as a key mechanism matching perfusion to metabolic demand [10]. Moreover, there is evidence that functional sympatholysis is not only present during exercise but can persist into the early recovery phase. In a human study using a cold-pressor test to evoke sympathetic vasoconstriction, forearm vascular conductance remained blunted in the first 10 minutes post-exercise compared to rest, indicating that vasoconstrictor responsiveness remains reduced for a time even after contractions have ceased [47]. In the context of overpacing, this asymmetric reoxygenation pattern provides a plausible mechanistic explanation for the frequently reported inability to re-establish a stable work rate after an excessively fast start. Even when external workload is reduced, the continued deoxygenation of previously inactive or intermittently active muscle groups may impose renewed metabolic stress upon re-engagement, thereby perpetuating a state of systemic overload.

By capturing these intensity-dependent differences between muscles, the developed method overcomes the limitations of traditional recovery-based metrics. Its reliance on HRrel enables physiological normalization across individuals with different fitness levels, creating comparability that fixed time windows cannot achieve. The ability to detect changes associated explicitly with exceeding VT2 enhances the analytical resolution of SmO_2_ data. It provides a practical framework for examining how central and peripheral regulatory mechanisms shape muscle oxygenation during and after high-intensity exercise. Moreover, the method offers a reproducible way to compare active and inactive muscles, providing new insights into the coordinated regulation of blood flow and oxygen delivery. While the present study employed cycling as a controlled experimental model, the observed dissociation between active and inactive muscle reoxygenation is likely to be particularly relevant for multimodal exercise formats. In such settings, muscle groups frequently transition between active and inactive roles, which may exacerbate the physiological consequences of delayed reoxygenation following high-intensity efforts.

The identification and quantification of the overload loop represent a novel contribution to understanding SmO_2_ dynamics during highintensity exercise. The results show that the loop emerges only when systemic metabolic stress exceeds VT2, indicating that this phenomenon reflects a systemic regulatory threshold rather than a local muscular event. This adds a new dimension to the interpretation of SmO_2_ signals in exercise physiology and demonstrates the potential of combining local and systemic parameters to reveal complex physiological interactions. Given the widespread use of wearable NIRS devices in sports and clinical contexts, the method introduced in this study may offer practical applications for assessing exercise intensity, evaluating recovery behavior, and investigating sympathetic vascular regulation in healthy and clinical populations.

Importantly, the present findings suggest that the performance impairments associated with overpacing cannot be attributed solely to local metabolic fatigue or substrate depletion. Instead, they point toward a disturbance in the coordination of systemic vascular regulation and regional oxygen delivery that persists into early recovery. From an applied perspective, the overload loop highlights that recovery during high-intensity exercise is not purely time-dependent but intensity-dependent. Exceeding VT2 may induce physiological conditions under which brief reductions in external workload are insufficient to restore balanced muscle oxygenation, providing a potential explanation for the disproportionate performance collapse observed after overpacing.

## CONCLUSIONS

Overall, this study provides new evidence that exceeding VT2 induces a distinctive and quantifiable pattern of delayed reoxygenation in inactive muscle, captured by the overload loop. The mathematical method developed here enables this pattern to be statistically validated and compared across muscle groups, providing a tool for future studies examining muscle-specific responses to high-intensity exercise and the underlying mechanisms governing oxygen distribution and recovery. Beyond its methodological contribution, the identified overload loop provides a physiological framework that may help explain why, following excessive early intensity, recovery within the same exercise bout often fails, as commonly observed in overpacing during high-intensity functional exercise.
